# Psychometric Properties of the Child Behavior Checklist (CBCL) for Ages 6–18 to Identify Autism Spectrum Disorders (ASD) in a Turkish Parent Sample

**DOI:** 10.1007/s10803-024-06495-z

**Published:** 2024-07-26

**Authors:** Mahir Uğurlu, Esra Sözer Boz, Sedat Turgut

**Affiliations:** 1 Department of Special Education, Bartin University, Bartin, Turkey; 2 Department of Educational Measurement and Evaluation, Bartin University, Bartin, Turkey; 3 Department of Primary Education, Bartin University, Bartin, Turkey

**Keywords:** Child behavior checklist, Autism spectrum disorder, Psychometric evaluation, Partial credit model

## Abstract

The Child Behavior Checklist for ages 6–18 (CBCL/6–18) is broadly used for psycho-educational assessment in identifying children’s behavior problems in special education and psychology. However, the usefulness of the CBCL/6–18 in a Turkish sample still needs to be investigated. The current study aimed to investigate the psychometric properties of the measures of the CBCL/6–18 within a sample of Turkish parents. The psychometric evaluation includes item calibration using the Partial Credit Model (PCM). We analyzed data from 548 parents who have children with autism spectrum disorder. According to the PCM calibration, the results suggested that the Internalizing, Externalizing, and Total Problem subscales were unidimensional and showed local independence successfully. All subscales demonstrated adequate reliability, indicating that the scale distinguishes between children with different behavior problems. The subscales had varying item step ordering, meaning that transitions from one category to second by parent ratings are relatively straightforward. Some items with easy-to-define behavior problems, for example, Item 42 (constipated), were more likely to be endorsed by parents. Consequently, the CBCL/6–18 has adequate psychometric properties for accurately assessing problem behaviors in children based on parent ratings.

## Introduction

The Child Behavior Checklist (CBCL), a parent report checklist, measures various emotional and behavioral problems (Achenbach & Rescorla, 2000). The CBCL is widely used because it allows the gathering of information quickly to determine multiple symptoms in different age groups. Rescorla (1988) identified one of the factors that emerged in her analysis of the CBCL as autistic/bizarre. This finding has strengthened the idea that the CBCL can be implemented in the screening of children with suspected autism spectrum disorder (ASD). Accordingly, subsequent studies have begun to investigate the potential of the CBCL in identifying childhood and adolescent ASD. Some studies have provided evidence that the CBCL helps identify children with ASD (e.g., Ooi et al., 2011; Pandolfi et al., 2014; So et al., 2013).

The CBCL is designed to obtain statements from parents regarding their child’s problems and competencies. The 1991 version of the CBCL was normed for ages 4 to 18 (CBCL/4–18; Achenbach, 1991), and the 2001 revised version was normed for ages 6 to 18 (CBCL/6–18; Achenbach & Rescorla, 2001). The CBCL has been translated into more than 90 languages and has been used in numerous studies (Hartini et al., 2015).

### Co-occurring Emotional and Behavioral Disorders (EBD) in Children with ASD

ASD is classified in the Diagnostic and Statistical Manual of Mental Disorders (DSM-V) as a heterogeneous group of neurodevelopmental disorders characterized by persistent deficits in social communication and interaction accompanied by restricted, repetitive patterns of behavior, interests, or activities (American Psychiatric Association, 2013). Children with ASD constantly want to maintain their routines and engage in stereotypical behavior (Chebli et al., 2016; Jiujias et al., 2017). Hyperactivity, and attentional and behavioral problems are frequently observed in children with ASD (Lian et al., 2022). They may exhibit behaviors such as harming themselves and their surroundings, not accepting the rules, not following instructions, experiencing anger outbursts, screaming, yelling, and committing physical attacks (O’Connor & Kirk, 2008). At the same time, children with ASD may face situations such as difficulties in friendships, depression, anxiety, peer bullying, and loneliness (Helles et al., 2015; Neary et al., 2015; Spain & Blainey, 2015).

Children with an ASD commonly present with one or more co-occurring EBDs. Such may include specific disorders classified in the Diagnostic and Statistical Manual of Mental Disorders, Fifth Edition (American Psychiatric Association, 2013) and International Classification of Diseases-11th Edition (World Health Organisation, 2019). EBD assessment in children with ASD should be an ongoing, broad-based process that includes both child characteristics and contextual variables. The resulting data will inform a comprehensive intervention that will include skills training for youth and families and environmental supports and modifications (Matson et al., 2009). Identifying children’s behavioral and emotional risk and intervention services can ameliorate symptoms and decrease the likelihood of adverse outcomes (Lane & Menzies, 2005; Walker & Shinn, 2002). Unfortunately, many students with emotional or behavioral problems are not identified, therefore losing the critical opportunity for intervention. Developing strategies to identify and treat students with emotional and behavioral risk remains an important task for researchers in special education (Horwitz et al., 2003, 2007; Kataoka et al., 2002). EBD may cause unpleasant results when they are not diagnosed in childhood (Beard & Sugai, 2004). It may negatively impact children’s social and academic development. If interventions are not made for EBD, both the child and their environment will suffer (Glover & Albers, 2007), and the issues can persist into adulthood (Weikart, 1998). The intensity and continuity of EBD may vary depending on the situation, so they must be examined by considering their occurrence and frequency (Gimpel & Holland, 2003).

Individuals with ASD often experience additional health and behavior problems (Maskey et al., 2013). These individuals experience high levels of emotional and behavioral difficulties beyond the symptoms of ASD (Brereton et al., 2006), potentially showing emotional, social, peer-related, and behavioral problems (Goodman et al., 2010). Despite differences in definitions and measurements of emotional and behavioral problems, consistent results have been obtained using various measurement tools (Charman et al., 2015). Early detection and intervention to minimize the effects of EBDs in children with ASD increases the probability of achieving positive results. For accurate determination, reliable and valid measurements should be used. This requires a wide variety of methods and measurements that are psychometrically valid and developmentally sensitive. Although more studies are needed, empirical studies support the CBCL/6–18 as one of the best-studied EBD rating scales in individuals with ASD (Pandolfi & Magyar, 2014).

### Child Behavior Checklist for Ages 6–18 (CBCL/6–18)

Researchers and clinicians should regularly check for possible behavioral and emotional symptoms in various contexts. Rating scales and behavioral checklists are helpful and practical tools for detecting a child’s symptoms. They can be used for screening and well-informed diagnosis (Lempp et al., 2012). The Achenbach System of Empirically Based Assessment is widely used to determine dimensional psychopathology in school-age children (Achenbach & Rescorla, 2001). The Child Behavior Checklist/Ages 6–18 (CBCL) is one of the most frequently used dimensional instruments for screening and diagnosing emotional and behavioral problems in children (Braet et al., 2011). The CBCL, which is based on observation of children in necessary situations, comprises different versions of child, parent, and teacher instruments, according to the child's age, to allow for multi-informative assessment (Achenbach & Rescorla, 2007).

Parent-report behavioral rating scales are generally used instead of comprehensive diagnostic interviews owing to their relative brevity and cost-efficiency. The purpose of the CBCL is to gather parent reports regarding their children’s problems and competencies (Dumenci et al., 2004). Using parent reports on the previous six months, it evaluates 118 different emotional and behavioral problems (Berube & Achenbach, 2004). The CBCL includes a correlated eight-factor structure of syndromes that are designated as follows: Anxious/Depressed, Withdrawn/Depressed, Somatic Complaints, Social Problems, Thought Problems, Attention Problems, Rule-Breaking Behavior, and Aggressive Behavior (Achenbach & Rescorla, 2001).

The CBCL is a parent report measure that evaluates observed functioning in internalizing and externalizing symptom domains. It offers scores along broad-band scales and narrow-band syndrome scales that were empirically developed and derived through factor analysis (Achenbach & Rescorla, 2001). The CBCL, a reliable and cost-effective parent-rated measurement tool for children and adolescents, is an empirically derived rating scale constructed through a series of quantitative analyses to determine the overlap of behavioral traits to capture specific dimensions of psychopathology. It has shown excellent reliability and validity in clinical and non-clinical populations (Pauschardt et al., 2010).

The CBCL measures emotional and behavioral problems in children and can be used as a screening tool for ASD in clinical settings (Achenbach & Rescorla, 2013). It is being used in an increasing number of studies on children with ASD. It has mainly been used to evaluate the types and correlates of emotional and behavioral problems in children with ASD (González & Stern, 2016; Hirata et al., 2016; Ross & Cuskelly, 2006; Samson et al., 2015; Son et al., 2015; Uğurlu & Eratay, 2022; Xu et al., 2014; Wade et al., 2014). The CBCL distinguishes behaviors and characteristics related to ASD well, accurately, and successfully (Matson & Cervantes, 2014). It is practical and effective in distinguishing the Withdrawn/Depressed, Social Problems, and Thought Problems syndrome scales between ASD and non-ASD children of school age (ages 6–18) (Biederman et al., 2010; Ooi et al., 2010). At an item level, the study found ten items were most predictive of ASD: acting young, obsessions, daydreaming, preferring to be alone, being clumsy, repeating acts, speech problems, staring, behaving strangely, and being withdrawn (So et al., 2013). Another study revealed that nine items were most predictive: acts young, does not get along with other kids, fears specific animals and situations, prefers to be alone, nervous, repeats acts, has speech problems, behaves strangely, and is withdrawn (Ooi et al., 2010).

### The Present Study

Some studies have examined the psychometric properties of the CBCL/6–18 scores among different samples (cultures or different groups) (Pandolfi et al., 2012; Penelo et al., 2017; Seleem et al., 2023). Most of the studies used Confirmatory Factor Analysis (CFA) or Exploratory Factor Analysis (EFA) in the Structural Equation Modeling (SEM) framework to test psychometric properties, such as factor structures across different groups. The factor structure of the Turkish version of the CBCL/6–18 was validated by using the CFA approach (Dumenci et al., 2004). Another framework, Item Response Theory (IRT), also has different approaches to test the psychometric properties of the measurement instruments. The purpose of the study was mainly to verify the functioning of the rating scale categorization of the CBCL/6–18 by the Turkish parent sample. The Partial Credit Model (PCM; Wright & Masters, 1982) was utilized to examine the psychometric properties of the CBCL/6–18 items among children with ASD based on parent ratings. The PCM can be considered an extension of the one-parameter logistic model (1PL) in the IRT framework and has Rasch model features such as person-level and item-level parameters (Embretson & Reise, 2000).

SEM approaches (e.g., CFA, EFA, etc.) focus on the relationships between observed variables and latent factors, testing predefined factor structures, theory, or measurement invariance among groups (Brown, 2015). However, PCM focuses on individual item responses and their ordered categories, modeling the probability of each response. PCM helps understand the functioning of items and improves measurement precision (Embretson & Reise, 2000). If the researchers want to determine how well each item discriminates between different ability levels, PCM is useful for designing and analyzing measurement instruments.

The PCM is appropriate for analyzing attitude or personality trait items that are polytomously scored with varying category scores (e.g., 0, 1, 2..). The response categories of the CBCL/6–18 have ordinal responses (i.e., 0, 1, and 2) indicating the levels of children’s behavior problems by parent ratings. PCM was employed to evaluate the probability of a behavior problem being rated based on children’s behavior levels (person ability) and the intensity of the behavior problem that the item is designed to measure (item difficulty). PCM analysis allows for a thorough examination of alignment with children’s behavior levels and parent ratings, as well as the creation of accurate measures that reflect the underlying construct of behavior problems.

The aim of this study was to investigate the psychometric properties of the subscale scores (internalizing, externalizing, and total problem) of the CBCL/6–18 across Turkish parents by applying the PCM. We assessed assumptions of the PCM (i.e., unidimensionality and local independence), reliabilities of the subscale scores, and item-person map. The research questions are as follows:

1. To what extent do the CBCL/6–18 subscales meet the assumptions of the PCM?
2. What are the reliabilities of the CBCL/6–18 subscales?
3. What are the step difficulty (category intersection) levels of the CBCL/6–18 subscales?

## Method

### Participants

The study participants comprised 548 parents, 313 females (57%) and 235 males (43%). The ages of the participants ranged from 29 to 53. 124 parents reported for their daughter and 424 for their son. The ages of these children range from 7 to 14. Seventy-six (14%) of these students have multiple disabilities.

### Measures and Data Collection

Children’s behavior problems with ASD were assessed by parent ratings using the CBCL/6–18. Data were collected through the CBCL/6–18 scale developed by Achenbach and Rescorla (2001) and adapted to Turkish by Erol and ve Şimşek (2010). The scale, consisting of 113 items, is graded as 0 (not true), 1 (somewhat true), and 2 (very true) according to the frequency of problem behaviors in the last six months. The scale has eight sub-factors: Anxious/Depressed (includes 16 items), Withdrawn/Depressed (includes 8 items), Somatic Complaints (includes 3 items), Social Problems (includes 11 items), Thought Problems (includes 10 items), Attention Problems (includes 26 items), Other Problems (includes 7 items), Rule-Breaking Behavior (includes 12 items), and Aggressive Behavior (includes 20 items). While each subscale can be scored separately, a total score can be obtained from the scale. We investigated psychometric properties for three subscales (i.e., internalizing, externalizing, and total problems) with 85 items.

In the data collection procedure, a meeting was held with the parents at the schools, and the purpose of the research was explained. Parents who volunteered to participate in the study were informed about the CBCL/6–18 scale. Then, the scale was implemented face-to-face.

### Data Analysis

#### Assumptions of Item Response Theory (IRT) Models

IRT models involve two key assumptions: unidimensionality and local independence (Embretson & Reise, 2000). Unidimensionality means that the model has a single ability for each examinee. Confirmatory Factor Analysis (CFA) was estimated using the Weighted Least Square Mean and Variance (WLSMV) to test the unidimensionality of eight sub-factors. The model-data fit is assessed with the Comparative Fit Index (CFI), the Tucker–Lewis Index (TLI), the Root Mean Square Error Approximation (RMSEA), and the Standardized Root Mean Square Residual (SRMR). A cutoff value for CFI/TLI > .90, RMSEA < .08, and SRMR < .10 are recommended for an adequate fit (Hu & Bentler, 1999). Yen (1984) proposed a simple test, Q_3,_ to check for any large violations of local independence. Q_3_ is computed as the linear correlation between the residuals, with critical values of item residual correlations > 0.20 indicating problematic.

Reliability was evaluated using different coefficients (i.e., Cronbach’s alpha (*α*), McDonald’s omega (*ω*), and ordinal alpha (Gadermann et al., 2012; Zumbo et al., 2007), which is for ordinal response data, with values greater than .70 indicating adequacy. Additionally, based on the item calibration, the person separation index (PSI; Wright & Masters, 1982) was estimated to evaluate how well the CBCL/6–18 separated children. Higher values (> .70) indicate that the CBCL/6–18 is suited to differentiate between children with different behavior problems.

#### Partial Credit Model

PCM was conducted for item calibration. When PCM is an extension of the Rasch model, the discrimination of an item is assumed to be equal for all items; thus, this term disappears from the model (Embretson & Reise, 2000). Assume that the item *i* is scored x = 0,…*m*_*i*_ with K_i_ = m_i_ + 1 response categories. The PCM specifies that the conditional probability that an examinee with latent ability *θ* obtains a category score x_*j*_,

$$Pix \left(\theta \right)=\frac{\text{exp}\sum_{j=0}^{x}(\theta -\delta ij)}{\sum_{r=0}^{mi}\text{exp}\sum_{j=0}^{r}(\theta -\delta ij)}$$

The parameter, δ_*ij*_, is called the step difficulty parameter (De Ayala, 2009). A δ_*ij*_ term can be directly interpreted as the point on the latent trait scale at which two consecutive category response curves intersect (Embretson & Reise, 2000). The category intersection parameters can be considered step difficulties associated with the transitions from one category to the next, and there are *m*_*i*_ step difficulties (intersections) for an item with *m*_*i*_ + 1 response categories. The CBCL/6–18 has ordinal response categories (0, 1, and 2), and we estimated two category intersection parameters (*β*, step difficulty) by applying PCM. These parameters indicate where, on the latent trait scale, the response of one category becomes relatively more likely than the previous category.

The unweighted (i.e., outfit) and weighted (i.e., infit) mean-square (MNSQ) estimates were employed to evaluate whether the items contribute efficiently to their own sub-factor. An item with an expected infit and outfit estimate of 1.0 would be ideal, indicating consistency between the data and the model. We considered the items as misfitting when outfit and infit statistics were below 0.5 or above 1.5 (De Ayala, 2009). If the infit and outfit values exceed 2.0, it indicates distortion or degradation to the item from the scale (Wright & Linacre, 1994). Violation of unidimensionality may result in item misfit. Thus, the validation of item fit also provides evidence for establishing unidimensionality.

An item-person map was constructed to evaluate the relationship between persons and items. It maps the range of children’s abilities or traits being measured (i.e., behavior problems) against the location of the item threshold on the same logic. This provides a useful way to determine if any items have disordered thresholds, which is an indicator that a higher category is not frequently used (Padgett & Morgan, 2020).

Linacre (2002) suggested that at least ten observations should be in each category for stable step calibrations in the Rasch model. Thus, frequencies of items 99 and 105 in the response category of 2 (very true) were zero, which was excluded from the analysis.

PCM statistical analyses were conducted with* R* (version 4.3.2) (R Core Team, 2023), using *eRm* (Mair & Hatzinger, 2007) and *mirt* (Chalmers, 2012) packages*.* M*plus* (version 8) (Muthén & Muthén, 1998–2012) was utilized for CFA.

## Results

### Unidimensionality and Local Independence

CFA was carried out to check the unidimensionality assumption for eight sub-factors. Table 1 shows the results of factor analysis. For the internalizing subscale, the results supported a one-factor solution separately in three sub-factors and a higher-order factor describing the internalizing sub-scale (χ^2^ (*df*) = 864.95 (290), CFI = .87, TLI = .86, RMSEA = .06, SRMR = .11). (Table 1).

**Table 1:** Model-data fit indices for CBCL/6–18 subscales from CFA

Subscales	Sub-factors	χ^2^ (*df*)	CFI	TLI	RMSEA	SRMR
Internalizing	Anxiety	225.6 (65)	.93	.92	.06	.10
	Somatic complaints	10.68 (5)	.91	.88	.04	.04
	Withdrawn	133.59 (20)	.93	.91	.10	.08
Externalizing	Rule-breaking problems	439.56 (77)	.92	.91	.09	.16
	Aggressive behavior	962.25 (135)	.91	.90	.10	.12
Total problem	Social problems	236.36 (44)	.87	.84	.08	.10
	Attention problems	177.02 (34)	.95	.93	.08	.06
	Thought problems	433.64 (87)	.88	.86	.08	.12

For the externalizing subscale, the factor loading of item 106 in the sub-factor of rule-breaking problems was below 0.20. Thus, this item was excluded from the subsequent analysis. The results supported a one-factor solution separately in two sub-factors describing the externalizing sub-scale (χ^2^ (*df*) = 1437.07 (458), CFI = .93, TLI = .92, RMSEA = .06, SRMR = .11). For the total problem subscale, the results supported a one-factor solution separately in two sub-factors and a higher-order factor describing the total problem sub-scale (χ^2^ (*df*) = 1714.57 (586), CFI = .93, TLI = .92, RMSEA = .06, SRMR = .11).

The local independence assumption was inspected with critical values for item residual correlations. Residual correlations of any item pair under three subscales were below .20 in magnitude, except for item 33 in the internalizing subscales, items 20 and 81 in the externalizing subscales, and item 70 in the total problem subscales, which were greater than .20. Thus, these items were excluded from the subsequent analyses. Local independence was established for these subscales.

### Reliability

The internalizing subscale reliabilities ranged from .70 to .72 for Cronbach α, .70 to.73 for McDonald ω, and .80 to .86 for ordinal alpha. The externalizing subscale reliabilities ranged from .77 to .89 for Cronbach α, .83 to .91 for McDonald ω, and .88 to .93 for ordinal alpha. The total problem subscale reliabilities ranged from .70 to .81 for Cronbach α, .70 to .84 for McDonald ω, and .78 to.86 for ordinal alpha. It can be concluded that the CBCL/6–18 subscales present with adequate levels of convergence between items.

PSI was estimated at .73 for internalizing subscale, .86 for externalizing, and .87 for the total problem. These findings suggested that the CBCL/6–18 subscales are suited to differentiate between children of different behaviors based on parent ratings.

### Item Calibration with PCM

Tables 2, 3, and 4 display item fit values and category intersections (step difficulties) of items for the internalizing, externalizing, and total problem subscales, respectively. For the internalizing subscale (see Table 2), infit (i.e., ranging from .83 to 1.21) and outfit MNSQ (i.e., ranging from .61 to 1.35) values for all items were between .5 and 1.5, indicating adequate model fit. This result is considered reasonable for defining children’s behavior observations by parent ratings.

**Table 2:** Item fit statistics for the internalizing subscales (25 items)

Item	Brief statements	Infit MNSQ	Outfit MNSQ	Category intersection	
				*β*_*1*_	*β*_*2*_
Anxiety/depressed					
14	Crying	1.04	1.02	.97	1.50
29	Fearful	1.08	1.13	1.22	.86
30	School fear	.88	.72	2.34	2.50
31	Bad things fear	.90	.70	2.83	2.39
32	Blameless	1.06	1.31	2.44	1.74
35	Unworthiness	.83	.62	2.43	2.72
45	Nervous	1.01	1.03	.41	1.30
50	Coward	.83	.80	.31	1.49
52	Sense of guilty	.88	.77	2.51	3.08
71	Anxious	.84	.68	1.97	1.99
91	Suicidality	.85	.61	3.76	1.54
112	Be apprehensive	.96	.86	2.29	2.21
Withdrawn					
5	Little interest	1.04	1.05	− .38	.98
42	Withdrawn	.95	.81	1.30	1.03
65	Does not speak	.97	.88	.94	.21
69	Does not share the secret	.88	.70	1.92	1.25
75	Shy	.94	1.00	.63	1.50
102	Little energetic	.96	.95	.55	1.04
103	Unhappy	.85	.75	1.26	2.37
111	Introvert	.82	.70	.94	1.04
Somatic complaints					
47	Have nightmare	1.21	1.35	1.00	1.08
49	Constipated	1.16	1.20	− .85	− .41
51	Dizziness	.90	.82	3.06	2.02
54	Fatigue	.98	.94	1.69	2.20
56	Aches	1.07	1.16	1.28	1.15

**Table 3:** Item fit statistics for the externalizing subscales (30 items)

Item	Brief statements	Infit MNSQ	Outfit MNSQ	Category intersection	
				*β*_*1*_	*β*_*2*_
Rule-breaking behavior					
2	Grumbling	1.14	1.30	− .63	.34
26	Lacks guilty	1.28	1.01	.29	.92
28	Not obey	.77	.71	.82	1.99
39	Bad friends	.85	.50	4.77	3.20
43	Cheating	.96	1.11	2.41	2.83
63	Big friends	1.33	2.65	–	–
67	Escape from home	.62	.56	.50	1.51
72	Starts fire	1.29	1.28	.73	1.96
73	Sexual problems	.91	.83	1.16	1.40
82	Robbery	1.01	1.06	3.51	3.13
90	Use bad language	1.02	.91	3.42	1.86
96	Sexual addiction	1.04	2.02	–	–
101	Escape from school	1.01	1.67	3.93	2.60
Aggressive behavior					
3	Arguing	1.04	.88	1.37	1.94
16	Abuse	.80	.73	1.78	2.45
19	Draws attention	1.25	1.41	.63	1.50
21	Hurts people	.78	.56	− .06	1.41
22	Disobedient	1.16	1.22	1.92	2.02
23	Disobedient in the school	.72	.69	− .96	1.27
37	Fights	.88	.68	− .23	1.80
57	Attacks people	.74	.59	.90	1.63
68	Screams	.70	.63	.76	1.48
86	Stubborn	.88	.88	− .35	1.03
87	Changes rapid mood	.95	.94	− .42	1.28
88	Offends	1.41	1.50	1.19	2.11
89	Suspects	1.11	1.46	2.50	2.27
94	Ridicules	.82	.59	1.97	2.09
95	Angry	.99	.99	1.06	1.68
97	Threats	.81	.51	3.23	2.78
104	Makes noise	.81	.68	1.01	1.55

**Table 4:** Item fit statistics for the total problem subscales (35 items)

Item	Brief statements	Infit MNSQ	Outfit MNSQ	Category Intersection	
				*β*_*1*_	*β*_*2*_
Social problem					
11	Dependent	.91	.90	.05	.78
12	Lonely	1.01	1.37	2.55	1.91
25	Not get along	.86	.80	.82	1.48
27	Jealous	1.32	1.49	.80	1.04
34	Out to get	.95	1.00	2.50	2.29
36	Get a hurt	.90	.80	1.17	2.05
38	Teased	.95	.94	2.30	2.27
48	Not liked	.97	.88	2.07	2.29
62	Clumsy	.94	.91	.81	.94
64	Prefer young	1.12	1.67	–	–
79	Speech problem	1.24	1.38	− .07	− .92
Thought problem					
9	Mind off	1.13	1.13	1.82	.67
18	Harm self	.95	1.19	2.88	1.21
40	Hears things	.94	.72	3.13	1.75
46	Twitch	.99	1.07	2.01	.94
58	Picks skin	.94	1.00	1.25	.87
59	Sleeps	1.11	1.15	1.19	1.52
60	Plays genitalia	.82	.79	− .30	1.18
66	Repeat acts	1.04	1.33	1.78	.01
76	Sleep loss	.95	.92	1.67	.72
83	Stores up	1.04	1.26	3.27	1.15
84	Strange behavior	1.12	1.29	2.40	.45
85	Strange ideas	.93	.86	2.38	.66
92	Sleepwalking	.91	.82	.69	.88
100	Sleep problem	.79	.71	.43	1.20
Attention problem					
1	Acts young	1.17	1.19	− .50	.14
4	Falls to finish	.84	.82	− .20	.43
8	Concentrate	.91	.93	− .98	.08
10	Sits still	.86	.81	.23	.57
13	Confused	.95	.94	.56	1.18
17	Daydream	1.07	1.28	1.11	1.79
41	Impulsive	.75	.75	− .05	.97
61	Poor school	.97	1.00	− .18	.86
78	Inattentive	.79	.78	− 1.22	− .18
0	Blank stare	.91	.89	− .02	1.09

Category intersection parameters were between −.85 and 3.76 logits for step one (*β*_*1*_), and between −.41 and 3.08 logits for step two (*β*_*2*_). For example, item 14 has ordered category intersection parameters (*β*_*1*_ = .97 and *β*_*2*_ = 1.5). This indicates that there is at least one trait level where every response option is most likely. Another example is that the category intersection parameters in item 29 are not ordered (*β*_*1*_ = 1.22 and *β*_*2*_ = .86). However, PCM models the ordered response categories and category intersections are not necessarily ordered (De Ayala, 2009). This does not imply that the definitions of the categories are disordered (Linacre, 2002). (Table 2).

For the externalizing subscale (see Table 3), infit (i.e., ranging from .70 to 1.41) and outfit MNSQ (i.e., ranging from .50 to 1.50) values for all items were between .50 and 1.50, indicating adequate model fit. However, two items had outfit MNSQ values greater than 1.50, including items 63 and 96 from the rule-breaking behavior sub-factor. This result may reflect noise in the data because the outfit MNSQ is sensitive to unexpected responses. Thus, these items were excluded from the subsequent analyses. Category intersection parameters were between − .96 and 4.77 logits for step one (*β*_*1*_), and between .34 and 3.20 logits for step two (*β*_*2*_). For example, item 2 has ordered category intersection parameters (*β*_*1*_ = − .63 and *β*_*2*_ = .34). Another example is that the category intersection parameters in item 39 are not ordered (*β*_*1*_ = 4.77 and *β*_*2*_ = 3.20). (Table 3).

For the total problem subscale (Table 4), infit (i.e., ranging from .75 to 1.32) and outfit MNSQ (i.e., ranging from .71 to 1.49) values for all items were between .50 and 1.50, indicating adequate model fit. However, item 64 from the social problem sub-factor had outfit MNSQ values greater than 1.50. This result indicates that it failed to define the same construct as the other items in the social problem sub-factor. Thus, this item was excluded from the subsequent analyses. Category intersection parameters were between -1.22 and 3.27 logits for step one (*β*_*1*_), and between − .92 and 2.29 logits for step two (*β*_*2*_). For example, item 11 has ordered category intersection parameters (*β*_*1*_ = .05 and *β*_*2*_ = .78). Another example is that the category intersection parameters in item 12 are not ordered (*β*_*1*_ = 2.55 and *β*_*2*_ = 1.91). Some items (for example, 34, 38, and 79) had similar disordered category intersections. (Table 4).

### Person-Item Map

**Fig. 1:**
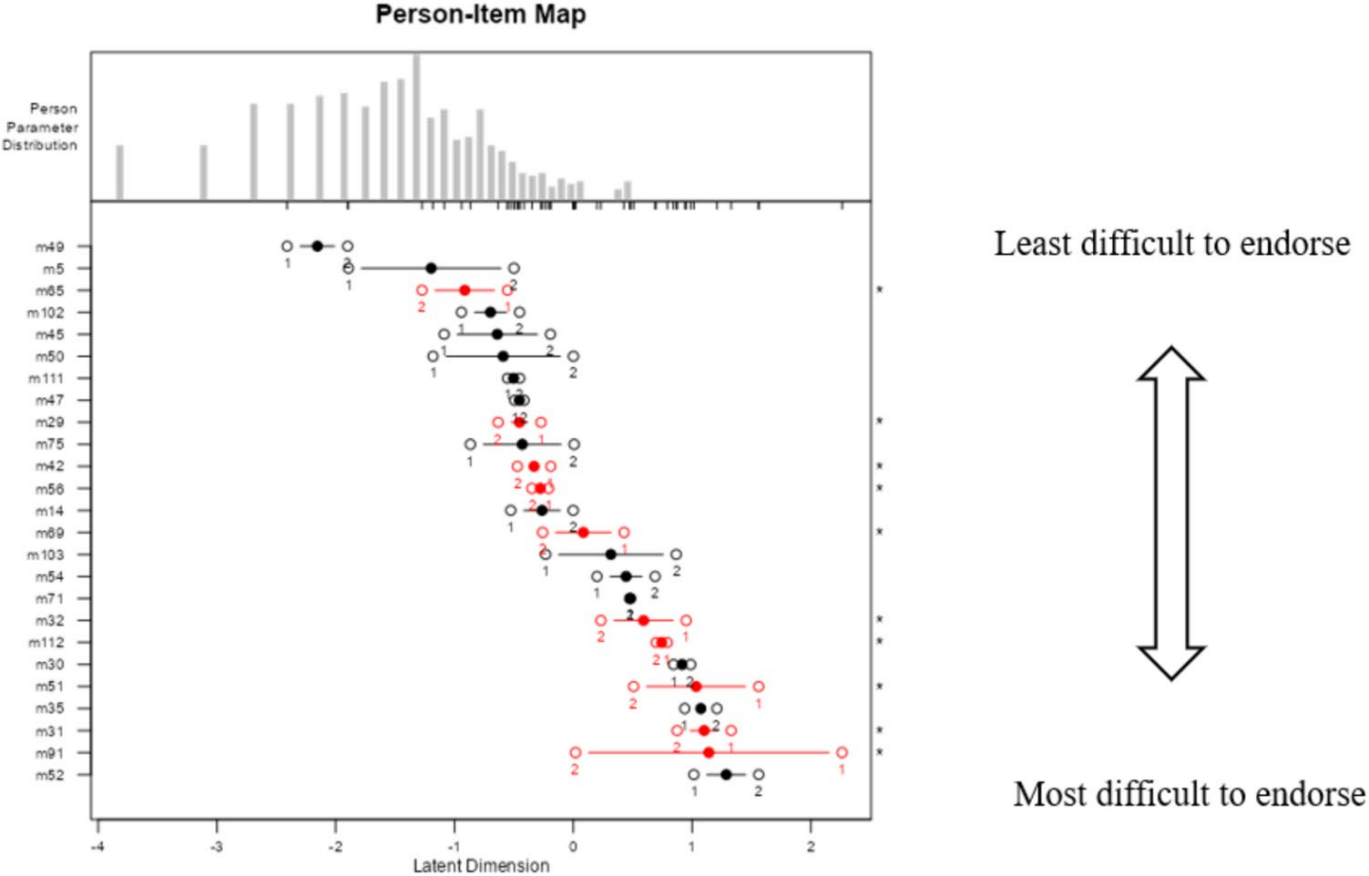
Person-item map for internalizing scores

**Fig. 2:**
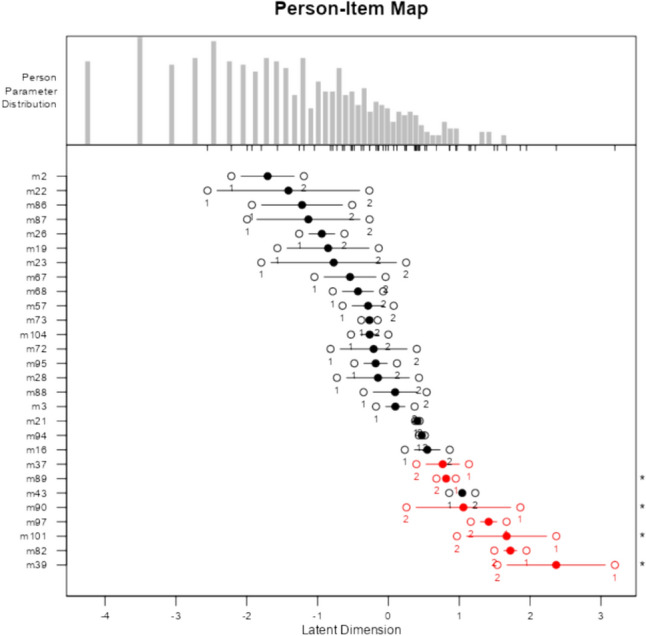
Person-item map for externalizing scores

**Fig. 3:**
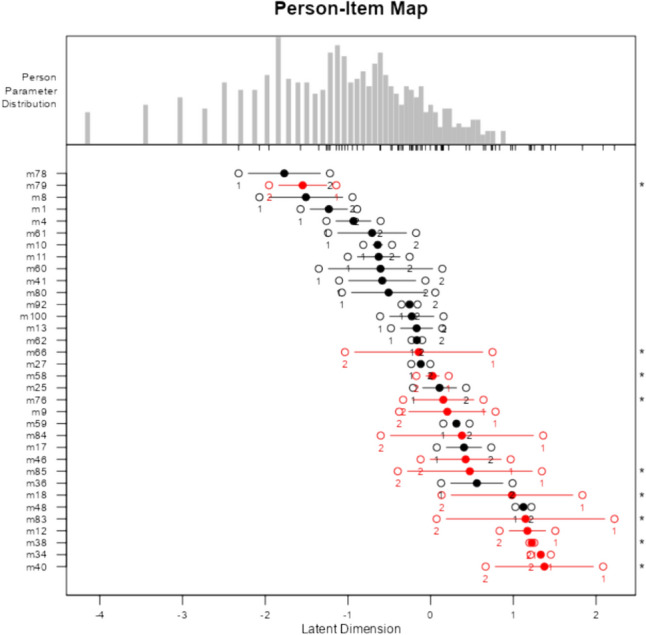
Person-item map for total problem scores

The person-item map displays the relationships of estimates for persons and items. These figures offer a useful representation of how the difficulty of items corresponds to the person parameters for the fitted PCM (Padgett & Morgan, 2020). The person-item map for internalizing items is shown in Fig. 1. It can be seen that category intersections are represented along with the item location. Items with disordered category intersections are marked with a (*) on the right vertical axis. Item-person maps for the other subscales are in the Appendix (Figs. 2 and 3). (Fig. 1).

Figure 1 displays the person-item map for internalizing items. The person parameter distribution was presented at the top of the figure. Items toward the top of the person-item map were more likely to be endorsed by parents, whereas those that were less likely to be endorsed by parents were located toward the bottom. Item 52 (sense of guilt), appearing at the bottom of the map, was the most difficult item to endorse for parents. Conversely, item 49 (constipated) at the top of the map was the easiest to endorse for parents. The marked (*) item (i.e., 65, 42, 56…) indicated having disordered category intersections. This information indicates that there is a need to examine the item (i.e., remove or revise the item).

## Discussion

Measurement and evaluation techniques provide researchers with various ways to examine the degree to which ordinal rating scales function in psychometrically meaningful ways. When the scale operates as expected, researchers can interpret ratings in the intended way, differentiate between categories, and compare ratings across individuals (Wind, 2023). The purpose of the study was to examine the psychometric properties of the CBCL/6–18 used for defining children with ASD within a sample of Turkish parents. PCM, which is one of the polytomous IRT models, was used for a detailed examination of the parent version of the CBCL/6–18 subscales (internalizing, externalizing, and total problem).

PCM results showed that misfit items were identified as two for externalizing and one for total problem subscales, excluding from subsequent analysis to improve the psychometric structures of the two subscales. After removing a total of three items, unidimensionality and local independence could be assumed for the remaining 25 items of internalizing, 28 items of externalizing, and 34 items for total problem subscales. Reliabilities of the CBCL subscales present with adequate levels of convergence (> .70) between items. High PSI (> .70) values indicate that the CBCL subscales are reliable for distinguishing between different behaviors of children with ASD.

One important contribution of this study is that it provides detailed information on the step difficulty of each item from the three subscales. It was found that category intersection parameters (step difficulties) for some items from the CBCL/6–18 subscales were disordered. Step disordering does not imply that the definitions of the categories are disordered (Linacre, 2002). This result can indicate that category 1 represents a narrow segment of the latent variable (behavior problem) or transitions from category 1 (somewhat true) to 2 (very true) by parent ratings are relatively easy. Disordering generally arises when the frequencies of category usage exhibit an irregular pattern. For example, item 51 (dizziness) from the internalizing subscale had the highest frequency for response category 0 (not true) and lowest frequency for category 2 (very true), resulting in step disordering. Thus, items with zero response categories (two items from the externalizing subscale) were excluded from the analysis. It seems that Turkish parents in this study might not be observing some problem behaviors as frequently.

The person-item map presents the range of person parameter distribution against the difficulty level of each item threshold on the same logistic scale. Among the internalizing items, items from the anxiety/depressed scale, for example, “sense of guilt” or “suicidality”, were less likely to be endorsed by parents (the most difficult items). One possible explanation is that parents could not recognize emotional disorders easily. Fewer difficult-to-endorse items (more likely to be endorsed by parents) were from the withdrawn scale; for example, “do not speak” and “little energetic”. The most difficult externalizing items were from the rule-breaking behavior scale, including “use bad language”, “cheating”, “bad friends”, which were behaviors less frequently observed by parents. The most difficult total problem items were from social and thought problem scales; for example, “hears things”, “teased”, “lonely”, which were individual-specified behaviors less likely to be observed by parents.

Another important contribution is that we can examine all the CBCL/6–18 subscales. However, the CBCL/6–18 is a widely used standardized parent rating scale, to our knowledge, this was the first study to examine the psychometric properties of the parent rating version of the CBCL/6–18 subscales in the Turkish parent sample. Researchers tested the versions of the CBCL (different age groups or parent/teacher forms) in different cultures. Ivanova et al. (2007) studied the factor structure of parent ratings of the CBCL/6–18 in 30 societies, including Turkey, and the results support the use of the scale in diverse societies. Al-Hendawi et al. (2016) confirmed factor structures of the CBCL/6–18 in the sample of Qatar, and Seleem et al. (2023) did the same in a sample of Egyptian children. Hsiao et al. (2023) examined the psychometric properties of the CBCL/1½–5 from low-income families using Rasch analysis. Another study by Pandolfi et al. (2012) examined the psychometric properties of the parent or caregiver rating of the CBCL/6–18 in a sample of youth with ASD. This study is one of the studies to test the scale's factor structure and to examine sensitivity to item response categories to distinguish children’s problem behaviors in a Turkish parent sample.

Early profiling of emotional and behavioral problems in children and adolescents offers better early detection and more timely and less expensive intervention (Braet & van Aken, 2006; Tick et al., 2007). To recognize children and adolescents with ASD, professionals need reliable and valid screening methods for behavioral and emotional problems. Nevertheless, screening through observation or interview is a significant burden, and these methods could be more reliable and cost-effective (Jensen & Weisz, 2002). Therefore, rating scales such as the CBCL/6–18 with clear cutoff points are essential for monitoring children with ASD with emotional and behavioral problems and selecting potential cases for further evaluation.

## Limitations

There were limitations to this study that need to be acknowledged, as well as directions for future research. The sample included children from 7 to 14 years, whereas the CBCL/6–18 is designed for ages 6 to 18. Future studies with the CBCL/6–18 extend its use in a larger and more heterogeneous sample to replicate the psychometric evaluations. The sample is also limited to generalizability to children from different age groups and backgrounds.

Another limitation is that this study only includes data from the parent observation ratings. The CBCL/6–18 is available for teacher ratings. Different versions of the CBCL ratings will enable us to examine each subscale for potential non-invariance items across parent and teacher ratings. Testing measurement invariance between different ratings (i.e., parent and teacher) will provide useful information to eliminate potential sources of bias and improve agreement between teacher and parent ratings. We recommended that more research on the CBCL/6–18 is needed to improve usability practically and support inferences from measures.

## Conclusion

The current study provides validity evidence for the factor structure of the CBCL/6–18 based on parent-ratings. The results indicate that the CBCL/6–18 subscales -internalizing, externalizing, and total problem- meet the assumptions of the PCM in a psychometrically satisfactory way. However, some items from different subscales are more difficult for parents to endorse. Overall, the psychometric evaluation shows that the CBCL/6–18 subscales are appropriate to use for detecting children’s behavior problems, especially children with ASD.

## Appendix

Item-person map for CBCL/6–18 subscales.

See Figs. 2 and 3.

## References

[CR1] Achenbach, T. M. (1991). *Manual for the Child Behavior Checklist/4-18 and 1991 profile*. University of Vermont.

[CR2] Achenbach, T. M., & Rescorla, L. A. (2000). *Manual for the ASEBA preschool forms and profiles*. University of Vermont.

[CR3] Achenbach, T. M., & Rescorla, L. A. (2001). *Manual for the ASEBA school-age forms & profiles*. University of Vermont, Research Center for Children, Youth, & Families.

[CR4] Achenbach, T. M., & Rescorla, L. A. (2007). *Multicultural supplement to the manual for the ASEBA school-age forms & profiles*. University of Vermont, Research Center for Children, Youth & Families.

[CR5] Achenbach, T. M., & Rescorla, L. A. (2013). Achenbach system of empirically based assessment. In F. Volkmar (Ed.), *Encyclopedia of autism spectrum disorders* (pp. 31–39). Springer.

[CR6] Al-Hendawi, M., Keller, C., & Cloninger, L. (2016). A psychometric analysis of the child behavior checklist for elementary school children in qatar. *Assessment for Effective Intervention,**41*(4), 220–229. 10.1177/1534508415626118

[CR7] American Psychiatric Association. (2013). *Diagnostic and statistical manual of mental disorders* (5th ed.). American Psychiatric Association.

[CR8] Beard, K. Y., & Sugai, G. (2004). First step to success: An early intervention for elementary children at risk for antisocial behavior. *Behavioral Disorders,**29*(4), 396–409.

[CR9] Berube, R. L., & Achenbach, T. M. (2004). *Bibliography of published studies using ASEBA instruments: 2004 edition*. University of Vermont, Research Center for Children, Youth, and Families.

[CR10] Biederman, J., Petty, C. R., Fried, R., Wozniak, J., Micco, J. A., Henin, A., Doyle, R., Joshi, G., Galdo, M., Kotarski, M., Caruso, J., Yorks, D., & Faraone, S. V. (2010). Child behavior checklist clinical scales discriminate referred youth with autism spectrum disorder: A preliminary study. *Journal of Developmental and Behavioral Pediatrics,**31*(6), 485–490. 10.1097/DBP.0b013e3181e56ddd 20585266 10.1097/DBP.0b013e3181e56ddd

[CR11] Braet, C., & van Aken, M. A. G. (2006). Developmental psychopathology: Substantive, methodological and policy issues. *International Journal of Behavioral Development,**30*(1), 2–4. 10.1177/0165025406059966

[CR12] Braet, C., Callens, J., Schittekatte, M., Soyez, V., Druart, C., & Roeyers, H. (2011). Assessing emotional and behavioural problems with the Child Behaviour Checklist: Exploring the relevance of adjusting the norms for the Flemish community. *Psychologica Belgica,**51*(3–4), 213–235. 10.5334/pb-51-3-4-213

[CR13] Brereton, A., Tonge, B., & Einfeld, S. (2006). Psychopathology in children and adolescents with autism compared to young people with intellectual disability. *Journal of Autism Developmental Disorders,**36*, 863–870. 10.1007/s10803-006-0125-y 16897401 10.1007/s10803-006-0125-y

[CR14] Brown, T. A. (2015). *Confirmatory factor analysis for applied research*. Guilford Press.

[CR15] Chalmers, R. P. (2012). mirt: A Multidimensional Item Response Theory Package for the R Environment. *Journal of Statistical Software,**48*(6), 1–29. 10.18637/jss.v048.i06

[CR16] Charman, T., Ricketts, J., Dockrell, J. E., Lindsay, G., & Palikara, O. (2015). Emotional and behavioural problems in children with language impairments and children with autism spectrum disorders. *International Journal of Language & Communication Disorders,**50*(1), 84–93. 10.1111/1460-6984.12116 25039810 10.1111/1460-6984.12116

[CR17] Chebli, S. S., Martin, V., & Lanovaz, M. J. (2016). Prevalence ofstereotypy in individuals with developmental disabilities: Asystematic review. *Review Journal of Autism and Developmental Disorders,**3*(2), 107–118. 10.1007/s40489-016-0069-x

[CR18] De Ayala, R. J. (2009). *The theory and practice of item response theory*. The Guilford Press.

[CR19] Dumenci, L., Erol, N., Achenbach, T. M., & Simsek, Z. (2004). Measurement structure of the Turkish translation of the Child Behavior Checklist using confirmatory factor analytic approaches to validation of syndromal constructs. *Journal of Abnormal Child Psychology,**32*(3), 335–340. 10.1023/b:jacp.0000026146.67290.0 15228181 10.1023/b:jacp.0000026146.67290.07

[CR20] Embretson, S. E., & Reise, S. P. (2000). *Item response theory for psychologists*. Lawrence Erlbaum Associates.

[CR21] Erol, N. ve Şimşek, Z. (2010). *Okul çağı çocuk ve gençler için davranış değerlendirme ölçekleri el kitabı [Handbook of behavior assessment scales for school-age children and adolescents]*. Mentis Yayıncılık.

[CR22] Gadermann, A. M., Guhn, M., & Zumbo, B. D. (2012). Estimating ordinal reliability for Likert-type and ordinal item response data: A conceptual, empirical, and practical guide. *Practical Assessment, Research & Evaluation,*. 10.7275/n560-j767

[CR23] Gimpel, G. A., & Holland, M. L. (2003). *Emotional and behavioral problems of young children: Effective interventions in the preschool and kindergarten years.* Guilford Press.

[CR24] Glover, T. A., & Albers, C. A. (2007). Considerations for evaluating universal screening assessments. *Journal of School Psychology,**45*(2), 117–135. 10.1016/j.jsp.2006.05.005

[CR25] González, M. L., & Stern, K. (2016). Co-occurring behavioral difficulties in children with severe feeding problems: A descriptive study. *Research in Developmental Disabilities,**58*, 45–54. 10.1016/j.ridd.2016.08.009 27591974 10.1016/j.ridd.2016.08.009

[CR26] Goodman, A., Lamping, D. L., & Ploubidis, G. B. (2010). When to use broader internalising and externalising subscales instead of the hypothesised five subscales on the strengths and difficulties questionnaire (SDQ): Data from British parents, teachers and children. *Journal of Abnormal Child Psychology,**38*(8), 1179–1191. 10.1007/s10802-010-9434-x 20623175 10.1007/s10802-010-9434-x

[CR27] Hartini, S., Hapsara, S., Herini, S. E., & Takada, S. (2015). Verifying the indonesian version of the child behavior checklist. *Pediatrics International,**57*, 936–941. 10.1111/ped.12669 25925632 10.1111/ped.12669

[CR28] Helles, A., Gillberg, C. I., Gillberg, C., & Billstedt, E. (2015). Asperger syndrome in males over two decades: Stability and predictors of diagnosis. *Journal of Child Psychology and Psychiatry,**56*(6), 711–718. 10.1111/jcpp.12334 25283685 10.1111/jcpp.12334

[CR29] Hirata, I., Mohri, I., Kato-Nishimura, K., Tachibana, M., Kuwada, A., Kagitani-Shimono, K., Ohno, Y., Ozono, K., & Taniike, M. (2016). Sleep problems are more frequent and associated with problematic behaviors in preschoolers with autism spectrum disorder. *Research in Developmental Disabilities,**49–50*, 86–99. 10.1016/j.ridd.2015.11.002 26672680 10.1016/j.ridd.2015.11.002

[CR30] Horwitz, S. M., Gary, L. C., Briggs-Gowan, M. J., & Carter, A. S. (2003). Do needs drive services use in young children? *Pediatrics,**112*, 1373–1378. 10.1542/peds.112.6.1373 14654612 10.1542/peds.112.6.1373

[CR31] Horwitz, S. M., Kelleher, K. J., Stein, R. E., Storfer-Isser, A., Youngstrom, E. A., Park, E. R., Heneghan, A. M., Jensen, P. S., O’Connor, K. G., & Hoagwood, K. E. (2007). Barriers to the identification and management of psychosocial issues in children and maternal depression. *Pediatrics,**119*(1), e208–e218. 10.1542/peds.2005-1997 17200245 10.1542/peds.2005-1997

[CR32] Hsiao, Y. Y., Qi, C. H., Dale, P. S., Bulotsky-Shearer, R., & Wang, Q. (2023). Measuring behavior problems in children from low-income families: A rasch analysis of the child behavior checklist for ages 1½-5. *Journal of Psychoeducational Assessment,**41*(5), 526–541. 10.1177/07342829231162216

[CR33] Hu, L. T., & Bentler, P. M. (1999). Cutoff criteria for fit indexes in covariance structure analysis: Conventional criteria versus new alternatives. *Structural Equation Modeling,**6*(1), 1–55. 10.1080/10705519909540118

[CR34] Ivanova, M. Y., Achenbach, T. M., Dumenci, L., Rescorla, L. A., Almqvist, F., Weintraub, S., Bilenberg, N., Bird, H., Chen, W. J., Dobrean, A., Döpfner, M., Erol, N., Fombonne, E., Fonseca, A. C., Frigerio, A., Grietens, H., Hannesdóttir, H., Kanbayashi, Y., Lambert, M., & Verhulst, F. C. (2007). Testing the 8-syndrome structure of the Child Behavior Checklist in 30 societies. *Journal of Clinical Child and Adolescent Psychology,**36*(3), 405–417. 10.1080/15374410701444363 17658984 10.1080/15374410701444363

[CR35] Jensen, A. L., & Weisz, J. R. (2002). Assessing match and mismatch between practitioner-generated and standardized interview-generated diagnoses for clinic-referred children and adolescents. *Journal of Consulting and Clinical Psychology,**70*(1), 158–168. 10.1037/0022-006X.70.1.158 11860042

[CR36] Jiujias, M., Kelley, E., & Hall, L. (2017). Restricted, repetitivebehaviors in autism spectrum disorder and obsessive-compulsive disorder: A comparative review. *Child Psychiatryand Human Development,**48*(6), 944–959. 10.1007/s10578-017-0717-0 10.1007/s10578-017-0717-0 28281020

[CR37] Kataoka, S. H., Zhang, L., & Wells, K. B. (2002). Unmet need for mental health care among U.S. children: variation by ethnicity and insurance status. *The American Journal of Psychiatry,**159*(9), 1548–1555. 10.1176/appi.ajp.159.9.1548 12202276 10.1176/appi.ajp.159.9.1548

[CR38] Lane, K. L., & Menzies, H. M. (2005). Teacher-identified students with and without academic and behavioral concerns: Characteristics and responsiveness. *Behavioral Disorders,**31*(1), 65–83. 10.1177/019874290503100103

[CR39] Lempp, T., de Lange, D., Radeloff, D., & Bachmann, C. (2012). The clinical examination of children, adolescents and their families. In J. M. Rey (Ed.), *IACAPAP e- Textbook of child and adolescent mental health* (pp. 1–25). International Association for Child and Adolescent Psychiatry and Allied Professions.

[CR40] Lian, X., Hin Hong, W. C., Xu, X., Zhu Kimberly, K., & Wang, Z. (2022). The influence of picture book design on visual attention of children with autism: A pilot study. *International Journal of Developmental Disabilities,**69*(6), 946–956. 10.1080/20473869.2022.2033590 37885844 10.1080/20473869.2022.2033590 PMC10599195

[CR41] Linacre, J. M. (2002). Optimizing rating scale category effectiveness. *Journal of Applied Measurement,**3*(1), 85–106. 11997586

[CR42] Mair, P., & Hatzinger, R. (2007). Extended Rasch Modeling: The eRm Package for the Application of IRT Models in R. *Journal of Statistical Software,**20*(9), 1–20. 10.18637/jss.v020.i09

[CR43] Maskey, M., Warnell, F., Parr, J. R., Couteur, A. L., & McConachie, H. (2013). Emotional and behavioral problems in children with an autism spectrum disorder. *Journal of Autism and Developmental Disorders,**43*, 851–859. 10.1007/s10803-012-1622-9 22895777 10.1007/s10803-012-1622-9

[CR44] Matson, J. L., & Cervantes, P. E. (2014). Commonly studied comorbid psychopathologies among persons with autism spectrum disorder. *Research in Developmental Disabilities,**35*(5), 952–962. 10.1016/j.ridd.2014.02.012 24629541 10.1016/j.ridd.2014.02.012

[CR45] Matson, J. L., LoVullo, S. V., Rivet, T. T., & Boisjoli, J. A. (2009). Validity of the autism spectrum disorder-comorbid for children (ASD-CC). *Research in Autism Spectrum Disorders,**3*(2), 345–357. 10.1016/j.rasd.2008.08.002

[CR46] Muthén, L. K., & Muthén, B. O. (1998–2012). *Mplus user's guide (7th Edition).* Muthén & Muthén.

[CR47] Neary, P., Gilmore, L., & Ashburner, J. (2015). Post-school needs of young people with high functioning autism spectrum disorder. *Research in Autism Spectrum Disorders,**18*, 1–11. 10.1016/j.rasd.2015.06.010

[CR48] O’Connor, K., & Kirk, I. (2008). Brief report: Atypical social cognition and social behaviours in autism spectrum disorder: Adifferent way of processing rather than an impairment. *Journal of Autism and Developmental Disorders,**38*(10), 1989–1997. 10.1007/s10803-008-0559-5 18712466 10.1007/s10803-008-0559-5

[CR49] Ooi, Y. P., Rescorla, L., Ang, R. P., Woo, B., & Fung, D. S. (2011). Identification of autism spectrum disorders using the Child Behavior Checklist in Singapore. *Journal of Autism and Developmental Disorders,**41*(9), 1147–1156. 10.1007/s10803-010-1015-x 20405192 10.1007/s10803-010-1015-x

[CR50] Padgett, R. N., & Morgan, G. B. (2020). Using the eRm package for Rasch modeling. *Measurement,**18*(3), 163–176.

[CR51] Pandolfi, V., & Magyar, C. I. (2014). Assessment of co-occurring emotional and behavioral disorders in youth with ASD using the Child Behavior Checklist 6-18. In V. B. Patel, V. R. Preedy, & C. B. Martin (Eds.), *Comprehensive guide to autism. *Springer.

[CR52] Pandolfi, V., Magyar, C. I., & Norris, M. (2014). Validity study of the CBCL 6–18 for the assessment of emotional problems in youth with ASD. *Journal of Mental Health Research in Intellectual Disabilities,**7*(4), 306–322. 10.1080/19315864.2014.930547 25419257 10.1080/19315864.2014.930547 PMC4239123

[CR53] Pandolfi, V., Magyar, C. J., & Dill, C. A. (2012). An initial psychometric evaluation of the CBCL 6–18 in a sample of youth with autism spectrum disorders. *Research in Autism Spectrum Disorders,**6*, 96–108. 10.1016/j.rasd.2011.03.009 22059091 10.1016/j.rasd.2011.03.009 PMC3207215

[CR54] Pauschardt, J., Remschmidt, H., & Mattejat, F. (2010). Assessing child and adolescent anxiety in psychiatric samples with the Child Behavior Checklist. *Journal of Anxiety Disorders,**24*(5), 461–467. 10.1016/j.janxdis.2010.03.002 20362414 10.1016/j.janxdis.2010.03.002

[CR55] Penelo, E., de la Osa, N., Navarro, J. B., Domenech, J. M., & Ezpeleta, L. (2017). The brief problem monitor-parent form (BPM-P), a short version of the child behavior checklist: Psychometric properties in Spanish 6- to 8-year-old children. *Psychological Assessment,**29*(11), 1309–1320. 28080108 10.1037/pas0000428

[CR56] R Core Team. (2023). *R: A Language and Environment for Statistical Computing*. Vienna, Austria. Retrieved from https://www.R-project.org/.

[CR57] Rescorla, L. (1988). Cluster analytic identification of autistic preschoolers. *Journal of Autism and Developmental Disorders,**18*(4), 475–492. 10.1007/BF02211868 3215877 10.1007/BF02211868

[CR58] Ross, P., & Cuskelly, M. (2006). Adjustment, sibling problems and coping strategies of brothers and sisters of children with autistic spectrum disorder. *Journal of Intellectual & Developmental Disability,**31*(2), 77–86. 10.1080/13668250600710864 16782592 10.1080/13668250600710864

[CR59] Samson, A. C., Hardan, A. Y., Lee, I. A., Phillips, J. M., & Gross, J. J. (2015). Maladaptive behavior in autism spectrum disorder: The role of emotion experience and emotion regulation. *Journal of Autism and Developmental Disorders,**45*(11), 3424–3432. 10.1007/s10803-015-2388-7 25711546 10.1007/s10803-015-2388-7

[CR60] Seleem, M. A., Amer, R. A., Elhosary, M., Saada, S., Hamza, E. A., Elfert, Y., Abdo, S. A. E., & Kabbash, & Achenbach, T. M. (2023). Psychometric properties and cross-cultural comparison of the Arabic version of the Child Behavior Checklist (CBCL), Youth Self Report (YSR), and Teacher’s Report Form (TRF) in a sample of Egyptian children. *Middle East Current Psychiatry*. 10.1186/s43045-023-00328-y

[CR61] So, P., Greaves-Lord, K., Van der Ende, J., Verhulst, F. C., Rescorla, L., & de Nijs, P. F. (2013). Using the child behavior checklist and the teacher’s report form for identification of children with autism spectrum disorders. *Autism,**17*(5), 595–607. 10.1177/1362361312448855 22914776 10.1177/1362361312448855

[CR62] Son, J. S., Zheng, L. J., Rowehl, L. M., Tian, X., Zhang, Y., Zhu, W., Litcher-Kelly, L., Gadow, K. D., Gathungu, G., Robertson, C. E., Ir, D., Frank, D. N., & Li, E. (2015). Comparison of fecal microbiota in children with autism spectrum disorders and neurotypical siblings in the Simons Simplex Collection. *PLoS ONE,**10*(10), e0137725. 10.1371/journal.pone.0137725 26427004 10.1371/journal.pone.0137725 PMC4591364

[CR63] Spain, D., & Blainey, S. H. (2015). Group social skills interventions for adults with high-functioning autism spectrum disorders: A systematic review. *Autism,**19*(7), 874–886. 10.1177/1362361315587659 26045543 10.1177/1362361315587659

[CR64] Team, R. C. (n.d.). The R project for statistical computing. Retrieved, June, 21, from https://www.r-project.org/

[CR65] Tick, N. T., van der Ende, J., & Verhulst, F. C. (2007). Twenty-year trends in emotional and behavioral problems in Dutch children in a changing society. *Acta Psychiatrica Scandinavica,**116*(6), 473–482. 10.1111/j.1600-0447.2007.01068.x 17997726 10.1111/j.1600-0447.2007.01068.x

[CR66] Uğurlu, M., & Eratay, E. (2022). The evaluation of emotional and behavioral disorders of children and adolescents affected by different deficiency disorders. *Ankara University Faculty of Educational Sciences Journal of Special Education,**23*(4), 873–891.

[CR67] Wade, J. L., Cox, N. B., Reeve, R. E., & Hull, M. (2014). Brief report: Impact of child problem behaviors and parental broad autism phenotype traits on substance use among parents of children with ASD. *Journal of Autism and Developmental Disorders,**44*(10), 2621–2627. 10.1007/s10803-014-2132-8 24805795 10.1007/s10803-014-2132-8

[CR68] Walker, H. M., & Shinn, M. R. (2002). Structuring school-based interventions to achieve integrated primary, secondary, and tertiary prevention goals for safe and effective schools. In M. R. Shinn, H. M. Walker, & G. Stoner (Eds.), *Interventions for academic and behavior problems II: Preventive and remedial approaches* (pp. 1–25). National Association of School Psychologists.

[CR69] Weikart, D. P. (1998). Changing early childhood development through educational intervention. *Preventive Medicine,**27*(2), 233–237. 10.1006/pmed.1998.0280 9579001 10.1006/pmed.1998.0280

[CR70] Wind, S. A. (2023). Detecting rating scale malfunctioning with the partial credit model and generalized partial credit model. *Educational and Psychological Measurement,**83*(5), 953–983. 10.1177/00131644221116292 37663538 10.1177/00131644221116292 PMC10470161

[CR71] World Health Organisation. (2019). *International classification of diseases* (11th ed.). World Health Organisation.

[CR72] Wright, B. D., & Linacre, J. M. (1994). Reasonable mean-square fit values. *Rasc Measurement Transactions,**8*, 370–371.

[CR73] Wright, B. D., & Masters, G. N. (1982). *Rating scale analysis*. Mesa Press.

[CR74] Xu, Y., Neece, C. L., & Parker, K. H. (2014). Parental depression and child behavior problems: A pilot study examining pathways of influence. *Journal of Mental Health Research in Intellectual Disabilities,**7*(2), 126–142. 10.1080/19315864.2013.787479

[CR75] Yen, W. M. (1984). Effects of local item dependence on the fit and equating performance of the three-parameter logistic model. *Applied Psychological Measurement,**8*(2), 125–145. 10.1177/014662168400800201

[CR76] Zumbo, B. D., Gadermann, A. M., & Zeisser, C. (2007). Ordinal versions of coefficients alpha and theta for likert rating scales. *Journal of Modern Applied Statistical Methods*. 10.22237/jmasm/1177992180

